# Surgical approach for high-energy posterior tibial plateau fractures

**DOI:** 10.4103/0019-5413.77131

**Published:** 2011

**Authors:** Shu-Qing Wang, You-Shui Gao, Jia-Qi Wang, Chang-Qing Zhang, Jiong Mei, Zhi-Tao Rao

**Affiliations:** Department of Orthopedics, Tongji Hospital, Tongji University, Shanghai, China; 1 Shanghai Sixth People’s Hospital, Shanghai Jiao Tong University, Shanghai, China

**Keywords:** Posterior tibial plateau fractures, surgical approach, posterolateral/posteromedial approach

## Abstract

**Background:**

High-energy fractures of posterior tibial plateau always need surgical treatment. Generally, posterior fragments of these fractures could not be exposed and reduced well in conventional anterior approaches. Although a posterolateral/posteromedial approach to manage posterior tibial plateau fractures can achieve satisfactory results, there are few presentations concerning the treatment of these high-energy injuries based on posterior approaches combined with anterior approach if necessary.

**Materials and Methods:**

Ten cases of posterior tibial plateau fractures from high-energy injuries were retrospectively reviewed and followed up for mean 26.5 months (range 14–45 months). A posterolateral/posteromedial approach was adopted primarily to fix main fragment in posterior tibial plateau, and intraoperative assessment of the stability of knee was done. An anterior approach was added if required.

**Results:**

Posterolateral approach was employed in seven cases, posteromedial in three, and additional anteromedial in three, and anterolateral in two cases. The average time to union of all 10 fractures was 3.7 months (range 3–5.5 months). Nine patients had satisfactory articular reduction. The range of motion of the knee averaged 2° of extension to 110.5° of flexion. No patient complained of knee instability. The average postoperative HSS score at the final followup was 92.70.

**Conclusions:**

High-energy fractures of posterior tibial plateau could be well treated based on posterior approaches combined with necessary anterior approach if required.

## INTRODUCTION

Although with the advent of sophisticated implants, ingenious surgical approaches, and precise imaging methods, it still presents great challenge to deal with high-energy fractures of the tibial plateau. These severe injuries could result in premature osteoarthritis, ligamentous injury, and lifelong pain and disability if restoration of the plateau surface and the axis of the leg could not be achieved.^1^–^3^ In general, Schatzker types IV–VI fractures were categorized as high-energy related, although it is believed this category system could not include all injury types now.^4^

The mechanism causing tibial plateau fractures is complex in high-energy injuries, probably coexisting with axial, varus and valgus, and rotational stress.^5^ Accordingly, the fractures appear complex in comparison with a single split or compression type. Except for fragment in posterior condyles, the articular surface in anterior usually appears comminuted and displaced. Additionally, meniscus and ligaments are often involved in primary injury.^6^ The knee instability will come out if these injuries are left untreated. The optimized treatment protocol should include assessing and reconstructing the stability apparatus in primary fixation of fractures. Obviously, it is not realistic to accomplish all these surgical aims in one single approach. Georgiadis used combined anterior and posterior approaches for the reduction and fixation of complex tibial plateau fractures involving a large split posteromedial fragment; as a result, all fractures united in good position with no significant complications and all patients had a good range of knee motion.^7^ And Carlson treated five patients with posterior bicondylar tibial plateau fractures by direct fracture exposure and fixation through posteromedial and posterolateral incisions.^8^ Two incisions are sometimes inevitable for exposure, reduction, fixation of fractures, and restoration of soft-tissue injuries.

In this study, we analyzed retrospectively 10 cases of high-energy posterior tibial plateau fractures which were managed based on a posterolateral/posteromedial approach. We hypothesize that the surgical technique is beneficial for the union of fractures and prevention of postoperative complications in posterior fractures of the tibial plateau. The surgical protocol for the reduction and biomechanical stabilization with minimally additional soft-tissue damage was discussed based on our experience.

## MATERIALS AND METHODS

Between January 2006 and January 2009, there were 78 consecutive patients with 78 tibial plateau fractures treated with open reduction and internal fixation (ORIF) at our institute in Orthopedic Department. Among these 78 fractures, 39 cases were caused by low-energy (Schatzker I-III) and were managed through an anterolateral (AL) approach, 10 were medial condylar fractures (Schatzker IV) and managed through an anteromedial (AM) approach, and 16 bicondylar fractures were treated via combined AL and AM approaches. The remaining 13 fractures were managed based on a posterior approach, and another anterior incision was employed in 8 cases.

In these 13 patients, 3 were lost. Basic demographic data and details including remaining 10 patients are shown in Table 1. There were seven men and three women with six right knees involved in and four in left. The majority of injuries (7 out of 10) were sustained in falling, and the other three were resulted from motor-vehicle accidents. All 10 fractures were closed; however, six had soft-tissue bruise and contusion. No patient had a compartment syndrome.

**Table 1:** Preoperative patient’s clinical details

Patients no.	Age (in year)	Gender	Mechanism	Side	AO/OTA code	Associated injury	Soft tissue damage
1	34	M	Falling	R	41-C2	ACL avulsion	Bruise/contusion
2	45	M	Falling	L	41-C1	None	None
3	25	F	Falling	R	41-B2	None	Bruise/contusion
4	41	M	Motor-veh. acc.	R	41-C2	None	None
5	44	M	Motor-veh. acc.	L	41-C1	None	None
6	47	F	Falling	L	41-C2	None	Bruise/contusion
7	49	M	Falling	R	41-B2	Lat. meniscus rupture	Bruise/contusion
8	76	F	Falling	L	41-B3	None	None
9	51	M	Falling	R	41-C1	Med. meniscus rupture	Bruise/contusion
10	27	M	Motor-veh. acc.	R	41-B2	None	Bruise/contusion

Preoperative treatment was centered on soft-tissue management, temporary fixation, and reduction of the fractures. Soft-tissue injuries including bruising and simple degloving could come to heal by dressing change. For the open wound with much oozing and suspicious infection, early ORIF could not be employed arbitrarily, especially when the incisions might cross the wound. We put the affected leg on a Brown’s frame and skeletal traction through calcaneal tubercle for assistant reduction. The weight of traction was about 5-8 kg. Complex and displaced fragments could partly come to reduction under the traction force, thus beneficial for intraoperative manipulation. It should be emphasized that a high incidence of compartment syndrome in these injuries need cautious evaluation and treatment, and pressure in the compartment should be measured if severe pain and paralysis of distal limb occurs.

### Operative procedure

**Figure 1:**
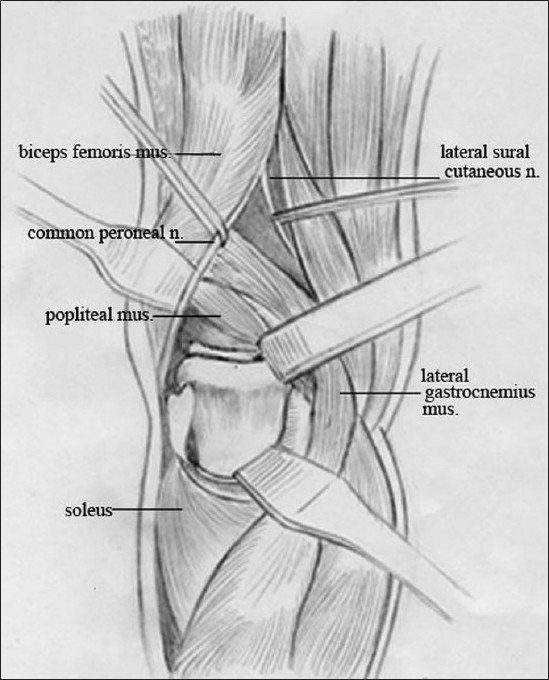
Line diagram of posterolateral approach (mus., muscle; n., nerve).

*Posterolateral (PL) approach*: The patient was placed in prone position, with injured lower extremity allowed to externally rotate and a high thigh tourniquet was employed. A skin incision about 8–10 cm long was made straight down along the medial border of the fibular head, starting about 2 cm above the popliteal crease. The skin and superficial fascia were incised by sharp dissection. The common peroneal nerve under the femoral biceps tendon was identified and protected before the deep fascia was opened. The femoral biceps and common peroneal nerve were retracted to lateral side to expose the popliteus. The middle genicular artery was ligated above the popliteus. The popliteus muscle above the fibular head was then retracted to medial, and the arcuate popliteal ligament and posterior capsule were incised. The insertion of soleus on the proximal fibula was dissected distally, not more than 5 cm below the articular level.^10^ The posterior ligamento-capsular complex was incised transversely then, and the meniscus was elevated for a careful examination of the fracture type [Figure 1].

**Figure 2:**
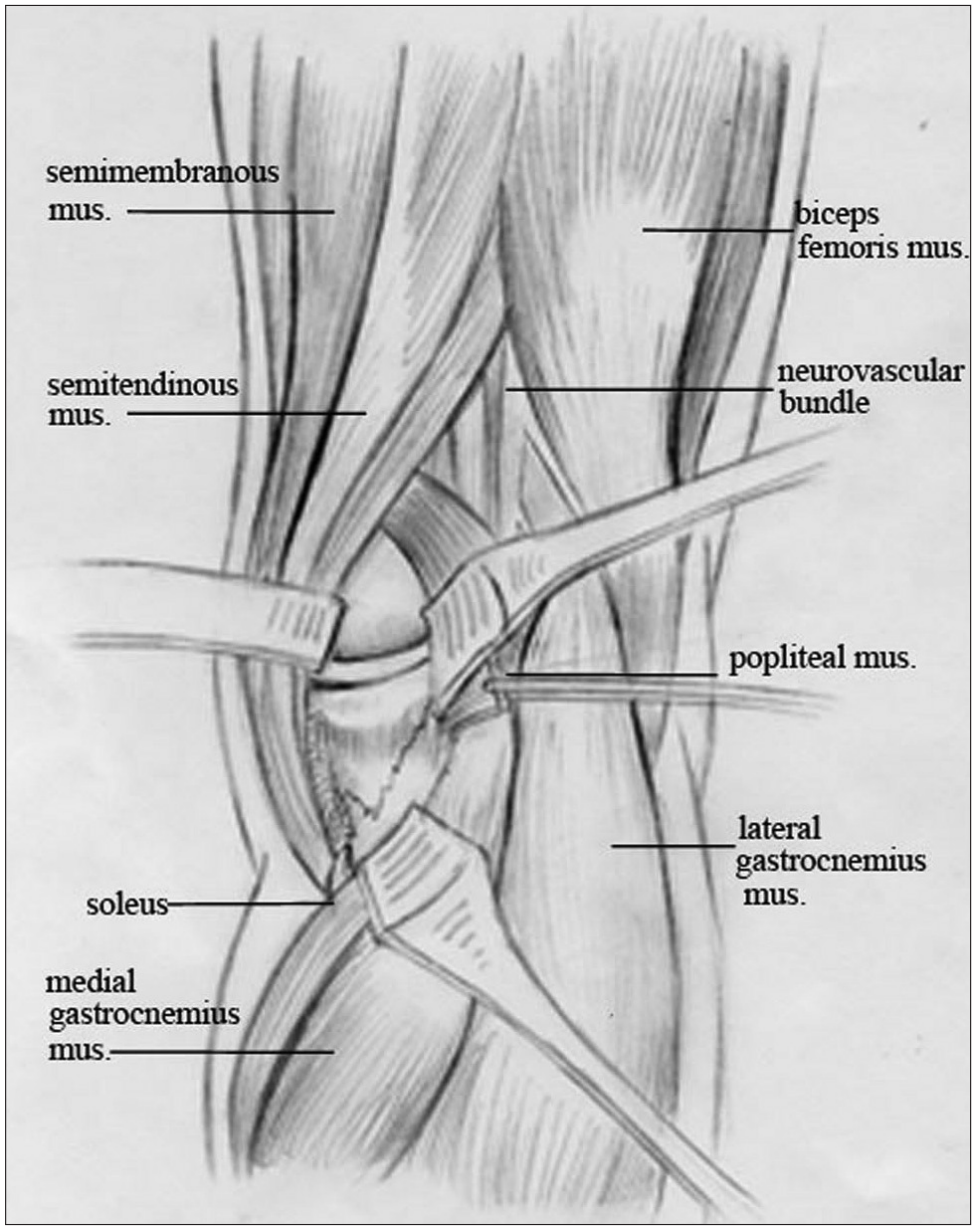
Line diagram of posteromedial approach (mus., muscle)

*Posteromedial (PM)* approach: The patient was placed in prone position. An S-shaped incision about 15–20 cm was made, which started approximately 1–2 cm posterior to the PM edge of the tibial metaphysis, paralleling the Satorius and posterior border of the pesanserinus tendon proximally. Full-thickness fasciocutaneous flaps were raised after identification and protection of the saphenous vein, medial sural cutaneous nerve, and common peroneal nerve. In the distal part of the incision, saphenous nerve and great saphenous vein were identified. The tendon of the medial head of the gastrocnemius was displayed with blunt dissection, and then divided leaving a stump for repair. The medial gastrocnemius was retracted to medial side, and the posteromedial back of the knee came into sight with the neurovascular bundle (popliteal vessels and tibial nerve) well protected. The popliteus and soleus origin could be elevated off from the PM aspect of the proximal tibia to lateral with a Hoffmann retractor as needed to obtain further exposure of the fracture [Figure 2].

**Figure 3:**
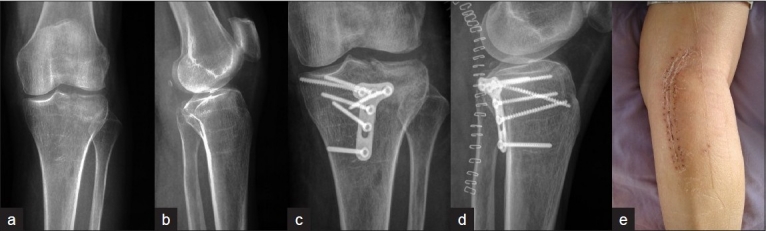
Anteroposterior (a) and lateral (b) radiographs showing the fragment of tibial plateau fracture locating posterolateral in Patient 5. Postoperative anteroposterior (c) and lateral (d) radiographs show satisfactory reduction and fixation through a direct PL approach. Clinical photograph of knee and leg, shows scar of PL approach (e). PL= Posterolateral

The reduction technique was similar in both PL and PM approaches. First, the articular surface was elevated through the fracture site under fluoroscopic guidance. Flexion of the knee to relax the posterior capsule could facilitate intraoperative reduction of the large fragments. Anteroposterior and lateral (oblique when needed) fluoroscopy was employed to assess the reduction quality. Second, the bone defects of metaphyseal could be filled with bone substitute (Osteoset^®^; Wright, Arlington, TN) or allograft (Aorui^®^, Taiyuan, Shanxi) after elevation of the depressed articular segments. They were employed in three patients, respectively. Third, 3.5-mm precontoured T(L)-plate and screw system was employed to fix the fractures, and a lag screw was used if the anterior segment could be well stabilized from posterior. This procedure was adopted in two cases. The wound was closed in routine way after repair of the muscle insertion and the deep fascia is left open. A deep drain was placed routinely [Figure 3a–e].

**Figure 4:**
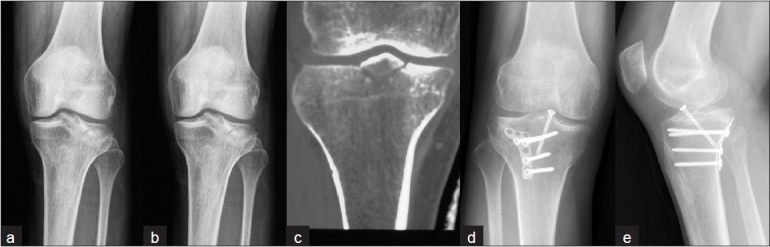
Anteroposterior (a) and lateral (b) radiographs of Patient 1 show the fracture of tibial plateau with main fragment in posterolateral, and CT scanning in coronary (c) shows avulsion fracture of the anterior cruciate ligament (ACL), which is a crucial instrument for knee stability. Postoperative anteroposterior (d) and lateral (e) views of the tibial plateau show satisfactory reduction and stabilization of the posterolateral fragments from PL approach and avulsion fracture from AM approach. PL= Posterolateral, AM = Anteromedial

**Figure 5:**
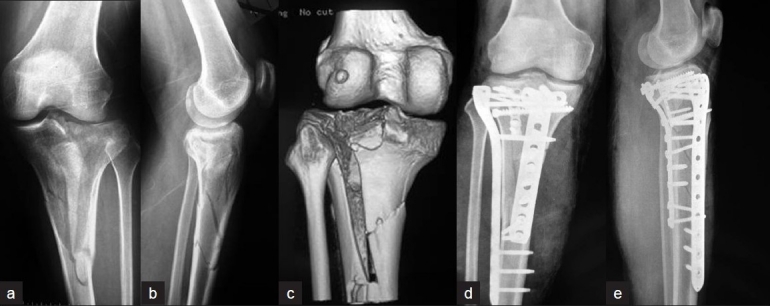
Anteroposterior (a), lateral (b) radiographs, and three-dimensional CT scan (c) of Patient 4 shows the complex fractures of the tibial plateau and metaphysis. Postoperative anteroposterior (d) and lateral (e) X-ray films show the fractures were well reduced and fixed via combined PM and AL approaches. PM= Posteromedial, PL= Posterolateral

After ORIF was finished posteriorly, the whole articular condition, knee stability, and tibial alignment were evaluated immediately. Five patients showed satisfactory reduction; however, three patients with an unsatisfactory articular surface in anterior and one with concomitant ACL avulsion and medial meniscus rupture individually needed further management [Figure 4a–e]. A conventional AL or AM approach was adopted to fix the fragments or repair the stabilization apparatus of the knee with patients in supine position. In principle, when a PL approach was adopted first, an AM approach was added; and an AL approach was added when first adoption was PM approach. The soft-tissue bridge between two incisions was wide enough to prevent deteriorating soft-tissue problems [Figure 5].

Postoperative management included intravenous antibiotics for 3 days, passive knee movement on a CPM, and active exercise with the help of surgeons. The partial weight bearing could be initiated at 3 weeks.

Two senior surgeons (S.Q.W.J.) assessed the quality of fracture reduction on the intraoperative and immediate postoperative plain radiographs of the knee including four radiographic parameters: they were articular reduction, sagittal alignment, coronal alignment, and condylar width.^9^ Agreement was reached by consensus. Each parameter was scored as satisfactory if within the following standard, articular reduction (≤2 mm step/gap), sagittal alignment (posterior proximal tibial angle 9±5°), coronal alignment (medial proximal tibial angle 87±5°), and condylar width (0–5 mm, inclusive).^9^ Reductions beyond these parameters were considered to be unsatisfactory. All numerical data were expressed as mean ± standard deviation, which were calculated by SPSS 15.0 (Chicago, IL).

## RESULTS

Totally, PL approach was employed in seven cases, PM approach in three, and additional anterior approaches were AM in three cases and AL in two. The average time from injury to ORIF was 5 days (range 1–7 days) and the average duration of followup was 26.5 months (range 14–45 months) after the operation. The average time taken for the surgery was 1 h (range 50 min to 1.5 h) in single posterior approach, and 1.5 h (range 1.3–2 h) in combined approaches. The average blood loss was 150 ml (range 100–300 ml).

The healing possess was determined both clinically and radiographically. The average time to union of all 10 fractures was 3.7 months (range 3–5.5 months). Nine patients had satisfactory articular reduction (≤2 mm step/gap) and one patient with an articular step of 4 mm. All patients demonstrated satisfactory coronal and sagittal alignment, and the condylar width was also within satisfactory standard.

Data concerning all 10 patients having clinical examination are summarized in Table 2. The range of motion (ROM) of the knee averaged 2.0° (range –5° to 15°) of extension to 110.5° (range 90°–125°) of flexion. No patient complained of knee instability. Physical examination did not reveal anterior or posterior, and varus or valgus instability of any affected knee. The average postoperative, hospital for special surgery knee score (HSS) at the final followup was 92.7 (range 88–98).

**Table 2:** Operative and followup details of patients

Patients no.	Time from injury to surgery (in days)	Approach	Postop. articular reduction	Followup (in months)	ROM (ext-flex)	Complications	HSS score
1	4	PL+AM	Anatomic	42	−2° to 120°	None	93
2	11	PM+AL	Anatomic	45	0°−100°	Superficial infection	90
3	4	PL+AM	Anatomic	22	0°−125°	None	98
4	5	PM+AL	Anatomic	28	−5° to 120°	None	91
5	7	PL	Anatomic	32	0°−110°	None	91
6	1	PL	Anatomic	14	5°−110°	Superficial infection	95
7	4	PM	Anatomic	35	−5° to 120°	None	96
8	5	PL	Imperfect	17	15°−100°	None	90
9	5	PL+AM	Anatomic	16	2°−110°	None	95
10	2	PL	Anatomic	14	10°−90°	None	88

PL = Posteriorlateral; PM = Posteromedial; AL = Anterolateral; AM = Anteromedial; ext-flex= Extension-flexion; Postop.= Postoperative

There were two patients suffering a sanguineous oozing from the wound postoperatively. Bacteria culture was negative and the incisions healed 3 weeks later with wound care. There were no other complication such as deep infection, incision necrosis, or the loosening and breakage of the implants.

## DISCUSSION

The mechanism concerning high-energy fractures of the tibial plateau is complex, and the particular types of injury are determined by multivariate including direction and magnitude of stress, knee position, and bone quality, etc. Apparently, high-energy fractures of tibial plateau are different from low-energy ones, which can be easily predicted according to their simple mechanism. Low energy usually leads to lateral split fractures (with possible compression), and articular surface maintains relatively intact that can be reduced and fixed through a single incision. Low-energy fractures of tibial plateau could be categorized as Schatzker type I–III. Several authors have pointed out that fractures of Schatzker type IV–VI are mostly caused by high energy; however, the Schatzker classification just limits to morphological assessment in sagittal plane and could not be used in coronal fractures of posterior condyles.^11^,^12^ There are only few clinical reports concerning high-energy fractures of tibial plateau until now, and it might be associated with complex mechanism of injury, difficult reduction and fixation, and unpredictable prognosis.

Anterior approaches are main method for treating tibial plateau fractures in the past decades. Even for single posterior condyle fractures, a plate-screw system or a lag screw is employed to fix the posterior fractures from anterior. This kind of osteosynthesis does not conform to the principle of biomechanics; meanwhile, it is hard to achieve the high standard of articular reduction, and flexion of the knee joint is not permitted for fear of fragment redisplacement. Although Lobenhoffer,^13^ Fakler,^12^ Tao,^14^ and Chang^10^ have illustrated different anatomic spaces in minor details to posterolateral and posteromedial condyles, the aim and essence is similar, that is, the expectation to expose, reduce, and fix the fragment. Compared with anterior approaches, the value of PM and PL approaches is in accordance with biomechanics, easy anatomical reduction of articular surface, which could be stabilized with antiglide or buttress plate.^15^–^17^ The concomitant injury of soft tissue always locates anterior; moreover, posterior soft tissue is thick and rich in blood supply. Good condition of posterior soft tissue allows an early operation, and postoperative problems of soft tissue are rare. Treatment protocol of tibial plateau fractures through posterior approaches is indicated in predominately posterior tibial plateau injuries, but as we know, anterior approaches still mean cardinal in the treatment of anterior fractures, ligament and meniscus injury, and metaphyseal comminution.^18^

When major fractures locate posterior condyles of tibial plateau, one PL or PM approach could be adopted for ORIF first. The evaluation of articular surface and knee stability is carried out during operation, which is determinant in whether anterior approaches should be added. Dual incisions are inevitable in fractures obviously displaced to anterior and posterior (burstlike), and proven ligament, meniscus injury needing reconstruction. As the fracture of posterior condyle is split-like and simple, ORIF could be started first in posterior with patient prone, and then, anterior approach is used with patient supine. Generally, combinations of PL and AM, PM and AL approaches are formed, which not only benefit for wide exposure, direct reduction, and fixation of fractures, but also for the wide soft-tissue bridge between two incisions, which could prevent skin necrosis.

The restoration of knee stability seems more important than the reduction of articular surface, although we could not approve Weigel and Marsh absolutely, who concluded that the knee joint cartilage appears to be tolerant of both the injury and mild-to-moderate residual articular displacement.^19^ However, several studies have proven that the knee instability is the most important factor for a poor prognosis. The varus and valgus deformity could be well prevented with maintaining the tibia in alignment in both sagittal and coronal planes, through precise preoperative planning, careful reduction, and biomechanical fixation. Soft-tissue injury, which is concomitant with plateau fractures, is quite common in high-energy trauma, and the interruption of surgery makes it more fragile. It is well demonstrated that complications of soft tissue after ORIF of tibial plateau fractures are notorious to deal with in past decades.^20^ As above-mentioned techniques of percutaneous and external fixations have lowered the rate of soft-tissue problems, however, they could not perform satisfactorily in articular comminution and instability. Posterior approaches are relatively complex in anatomy, but complications concerning soft tissue are rare. Soft-tissue complications in anterior approaches could be prevented through choosing suitable operation time, reasonable management of tissue around the cut, and early rehabilitation. In our cases of dual incisions, all soft-tissue problems appear anterior. Any suspicious infection including oozing or swelling should be cautiously managed in early stage.

Of course, the majority of tibial plateau fractures could achieve satisfactory results with a single anterior or posterior approach.^18^,^21^,^22^ Various external fixators or hybrid of external and internal fixation play a critical role when severe soft-tissue damage exists. The principles of staged treatment and individual characteristics proposed by Tscherne and Lobenhoffer^23^ should be noted if polytrauma or concomitant soft-tissue problems do not permit an optimized treatment. It could be concluded from the cases that soft-tissue complications in posterior approaches are fewer than those in anterior.

The limitation of this study is that the sample size is quite small. Although it was found that postoperative complications were low and short-term prognosis was satisfactory, we cautiously concluded that posterior approaches and necessarily combined anterior approach conceived of significant value for the management of tibial plateau fractures. On the prerequisite of excellent reduction and fixation, we incline to adopt a single posterior approach to deal with tibial plateau fractures, although the comparison between anterior and posterior approaches should be analyzed and expounded further.
